# Intramuscular 17-hydroxyprogesterone caproate to prevent preterm birth among HIV-infected women in Zambia: study protocol of the IPOP randomized trial

**DOI:** 10.1186/s12884-019-2224-8

**Published:** 2019-02-27

**Authors:** Joan T. Price, Bellington Vwalika, Bethany L. Freeman, Stephen R. Cole, Helen B. Mulenga, Jennifer Winston, Felistas M. Mbewe, Elwyn Chomba, Lynne M. Mofenson, Dwight J. Rouse, Robert L. Goldenberg, Jeffrey S. A. Stringer

**Affiliations:** 1 Division of Global Women’s Health, Department of Obstetrics and Gynecology, University of North Carolina at Chapel Hill, 3009 Old Clinic Building, Campus Box 7577, Chapel Hill, NC 27599-7577 USA; 2 Department of Obstetrics and Gynaecology, University of Zambia School of Medicine, Lusaka, Zambia; 3 Department of Epidemiology, University of North Carolina at Chapel Hill, Chapel Hill, NC USA; 4 Pharmaceutical Society of Zambia, Lusaka, Zambia; 5 UNC Global Projects – Zambia, Lusaka, Zambia; 6 Department of Paediatrics, University Teaching Hospital, Lusaka, Zambia; 7 Elizabeth Glaser Pediatric AIDS Foundation, Washington, DC USA; 8 Department of Obstetrics and Gynecology, Brown University, Providence, RI USA; 9 Department of Obstetrics and Gynecology, Columbia University, New York, NY USA

**Keywords:** Preterm birth, Progesterone, 17-alpha hydroxyprogesterone caproate, HIV, Antiretroviral therapy, Sub-Saharan Africa

## Abstract

**Background:**

Each year, an estimated 15 million babies are born preterm, a global burden borne disproportionately by families in lower-income countries. Maternal HIV infection increases a woman’s risk of delivering prematurely, and antiretroviral therapy (ART) may compound this risk. While prenatal progesterone prophylaxis prevents preterm birth among some high-risk women, it is unknown whether HIV-infected women could benefit from this therapy. We are studying the efficacy of progesterone supplementation to reduce the risk of preterm birth among pregnant women with HIV in Lusaka, Zambia.

**Methods:**

The Improving Pregnancy Outcomes with Progesterone (IPOP) study is a Phase III double-masked, placebo-controlled, randomized trial of intramuscular 17-alpha hydroxprogesterone caproate (17P) to prevent preterm birth in HIV-infected women. A total of 800 women will be recruited prior to 24 weeks of gestation and randomly allocated to 17P or placebo administered by weekly intramuscular injection. The primary outcome will be a composite of live birth prior to 37 completed gestational weeks or stillbirth at any gestational age. Secondary outcomes will include very preterm birth (< 34 weeks), extreme preterm birth (< 28 weeks), small for gestational age (<10th centile), low birth weight (< 2500 g), and neonatal outcomes. In secondary analysis, we will assess whether specific HIV-related covariates, including the timing of maternal ART initiation relative to conception, is associated with progesterone’s prophylactic efficacy, if any.

**Discussion:**

We hypothesize that weekly prenatal 17P will reduce the risk of HIV-related preterm birth. An inexpensive intervention to prevent preterm birth among pregnant women with HIV could have substantial global public health impact.

**Trial registration:**

NCT03297216; September 29, 2017.

## Background

Each year worldwide nearly 15 million babies are born prior to 37 weeks of gestation, of whom 1 million die as a consequence of prematurity [1]. The burden of preterm birth (PTB) and its associated mortality and long-term disability is disproportionately borne by the world’s poorest families. More than 60% of global preterm deliveries occur in South Asia and sub-Saharan Africa, where resources to care for premature newborns are scarce and case fatality is high [2]. The geographic disparity in rates of prematurity may in part reflect the distribution of maternal HIV, which increases the risk of PTB [3]. Of 1.5 million women living with HIV who become pregnant each year, the overwhelming majority reside in either sub-Saharan Africa (87%) or South Asia (5%) [4]. While expanding coverage of antiretroviral therapy (ART) among pregnant women living with HIV has drastically reduced the incidence of mother-to-child transmission, maternal ART exposure does not appear to ameliorate the increased risk of PTB in HIV-infected pregnant women [5–10]. Additionally, neonatal mortality remains elevated in HIV-infected pregnant women on ART compared to HIV-uninfected women; in a study comparing women on efavirenz or dolutegravir-based ART, neonatal mortality was 2.3% among HIV-infected compared to 1.4% among HIV-uninfected women [9]. A considerable proportion of this neonatal mortality appears to be secondary to PTB [6].

Prenatal progesterone reduces the risk of preterm delivery in women who have had a prior spontaneous PTB and in those with sonographic evidence of cervical shortening in the mid-trimester. It is standard of care in the United States for these indications [11]. A 2013 Cochrane meta-analysis of progesterone to prevent PTB among women reporting a prior PTB aggregated data from 10 randomized trials studying prenatal prophylaxis by the intramuscular (IM, *n* = 4 studies), vaginal (*n* = 5 studies), or oral (*n* = 1 study) route and estimated the risk ratio of birth prior to 37 weeks among women receiving active drug to be 0.55 (95% CI: 0.42, 0.74) [12].

We seek to determine whether 17-hydroxyprogesterone caproate (17P) will reduce the risk of PTB among HIV-infected pregnant women receiving ART. Here we present the study protocol for a randomized trial designed to answer this question.

## Methods

### Study design

**Fig. 1:**
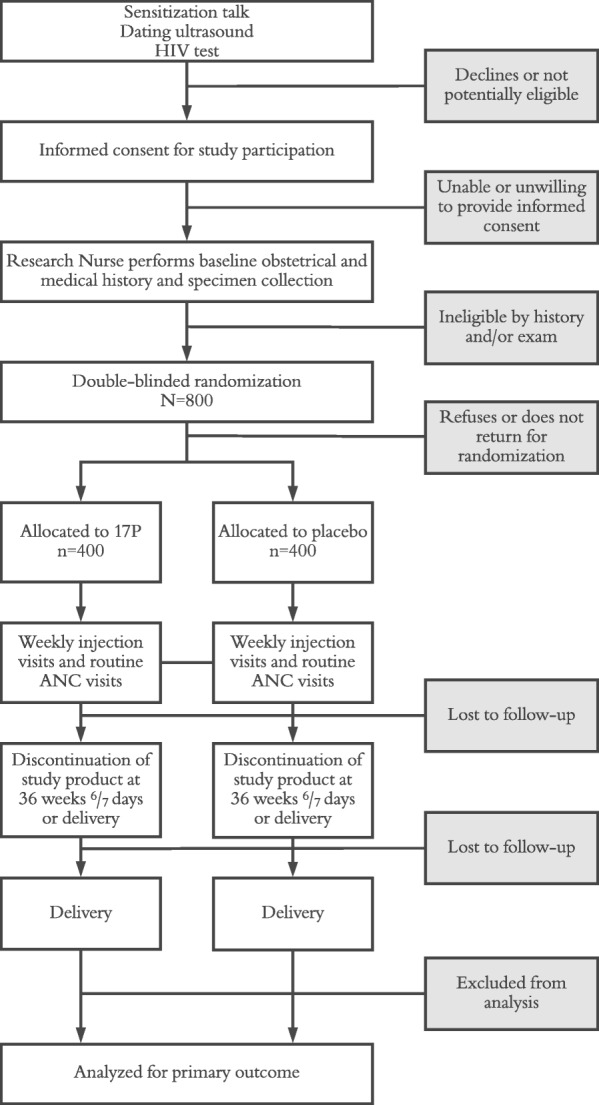
IPOP study participant flow diagram

The Improving Pregnancy Outcomes with Progesterone (IPOP) trial is a double-masked, placebo-controlled, randomized trial of 17P to prevent PTB among HIV-infected women in Zambia. It is registered with clinicaltrials.gov under identifier: NCT03297216. Participants are randomly assigned to weekly intramuscular administration of either 17P or placebo manufactured to be indistinguishable started between 16 and 24 weeks gestational age. The study’s primary outcome is a composite measure of delivery prior to 37 weeks or stillbirth at any gestational age. IPOP is being conducted in the antenatal clinics of the Kamwala District Health Centre (KDHC) and Women and Newborn Hospital of the University Teaching Hospital (WNH-UTH) in Lusaka. We also recruit participants from other public sector facilities in Lusaka. The IPOP trial has been designed following the Consolidated Standards of Reporting Trials (CONSORT 2010) Statement and the Standards for Protocol Items: Recommendations for Interventional Trials (SPIRIT 2013) (Fig. 1) [13, 14].

### Study participants

Women meeting the following inclusion criteria are eligible to participate in the IPOP study: (1) 18 years of age or older; (2) viable intrauterine singleton pregnancy confirmed by ultrasound; (3) less than 24 ^0^/_7_ weeks of gestation; (4) antibody-confirmed HIV-1 infection; (5) currently receiving ART or intending to commence ART in pregnancy; (6) ability and willingness to provide written informed consent; (7) intent to remain in current geographical area of residence for the duration of study; and (8) willing to adhere to weekly study visit schedule. We exclude from participation any woman with the additional exclusion criteria: (1) confirmed prior spontaneous preterm birth; (2) multiple gestation; (3) known uterine anomaly; (4) planned or in situ cervical cerclage; (5) major fetal anomaly detected on screening ultrasound; (6) indication for planned delivery prior to 37 weeks (e.g. prior classical cesarean); (7) evidence of threatened abortion, preterm labor, or ruptured membranes at time of enrollment; (8) known allergy or medical comorbidity listed as a contraindication to 17P in the prescribing information; (9) prior participation in the trial; and (10) any other condition (social or medical) which, in the opinion of the study staff, would make trial participation unsafe or complicate data interpretation.

Current Zambia treatment guidelines recommend ART initiation for all individuals living with HIV, including pregnant and breastfeeding women [15]. The current ART regimen for first-line therapy for pregnant women living with HIV in Zambia is combination tenofovir disoproxil fumarate, lamivudine and efavirenz, with second-line therapy being combination zidovudine, lamivudine and a protease inhibitor, lopinavir-ritonavir or atazanavir-ritonavir; most pregnant women are receiving first-line ART.

### Intervention

Trial participants are randomly allocated to one of two treatment groups in a 1:1 ratio. The first randomization group (17P) receives weekly 1 mL injections of 250 mg 17-hydroxyprogesterone caproate while the other group (control) receives placebo manufactured to be indistinguishable, also by injection.

Study product (both active drug and placebo) is produced by AMAG Pharmaceuticals of Waltham, MA, USA, under the brand name of Makena**®**. Packaged and labeled product is shipped to each study site and dispensed to the administering nurse by an on-site pharmacist or pharmacy technician. Pharmacy staff have no direct contact with participants or potential participants, and are the only staff in Zambia not masked to treatment allocation. Participants begin weekly administration of study product from the day of randomization (between 16 ^0^/_7_ and 23 ^6^/_7_ gestational weeks, inclusive) until 36 ^6^/_7_ gestational weeks, stillbirth or delivery, whichever is sooner.

If an adverse drug reaction is identified in any participant receiving injections of study product, staff will consult with investigators to determine whether the reaction requires temporary or permanent discontinuation of study product based on the severity of the event and whether or not the event resolves. Study product will be immediately and permanently discontinued for any participant who has an anaphylactic reaction. The investigator will decide whether to un-blind the treatment group to guide treatment and future care for the participant. In general, adverse drug reactions will typically not require un-blinding of study group and will not necessarily indicate that a participant is receiving the active product since components in the placebo base may also result in adverse reactions.

### Objectives

The primary objective of IPOP is to evaluate – among HIV-infected pregnant women receiving ART – whether 17P will reduce the risk of the composite outcome (live birth prior to 37 weeks of gestation or stillbirth at any gestational age) relative to placebo. Secondary objectives include: (1) to assess, through a subgroup analysis, whether 17P will reduce the risk of the primary outcome among women either (a) newly initiating ART or (b) continuing ART that was started prior to conception; (2) to assess the effect of timing of ART initiation on risk of the composite primary outcome and its components by comparing women newly starting ART to those who initiate ART prior to conception (a non-randomized comparison); and (3) to assess the extent to which exclusion of non-inflammatory PTB phenotypes from our outcome definition modifies the efficacy of 17P to prevent the composite primary outcome and/or any association between timing of ART initiation and the composite primary outcome. Finally, our tertiary objective is to investigate the underlying biology of PTB and other adverse pregnancy outcomes with particular attention to systemic (peripheral blood) inflammation and immune activation, local (vaginal) inflammation, and alterations of the vaginal microbiota.

### Study procedures

Our recruitment activities include community sensitization in the catchment area of the recruitment clinics to educate community members about the trial and encourage early presentation for antenatal care. In addition, study staff conduct health talks at the recruitment clinics focusing on the importance of early and complete antenatal care, possible prevention of preterm delivery, and study inclusion and exclusion criteria.

Study staff identify potential participants attending antenatal care prior to 24 estimated gestational weeks (Table 1). An ultrasound is conducted to confirm potential study eligibility and, among women between 16 and 24 weeks of gestation, cervical length by transvaginal ultrasound [16]. Those who meet preliminary eligibility by ultrasound and who agree to participation undergo a detailed informed consent process in English, Bemba, or Nyanja, depending on their language preference. After consent, study staff verify antenatal and HIV history data through a baseline questionnaire and review of participants’ available medical records, perform a physical exam, and complete point of care testing for antenatal care and to confirm participants’ HIV status.

**Table 1:** IPOP study schedule of evaluations

Visit Number	0.0	1.0	2.0	3.0	4.0	5.0	6.0	7.0
Gestational Age (weeks)	< 24	16–23	24	28	32	36	Delivery^c^	42 days
Administrative/regulatory procedures								
Informed consent	●							
Confirmation of eligibility		●						
Collection/review of locator info	●	●	●	●	●	●	●	●
Randomization		●						
Clinical/behavioral procedures								
Ultrasound^a^	●	●			●			
Obstetrical history	●							
Medical history and clinical exam^a^	●		●		●	●	●	●
Concomitant medication assessment	●		●		●	●	●	●
Demographic history	●							
Behavioral and nutritional assessment	●							●
Maternal depression screen	●							●
Infant clinical assessment							●	●
Study product procedures								
17P Adherence counseling^b^		●	●	●	●	●		
Study drug injections^b^		●	●	●	●	●		
Side effects assessment^b^			●	●	●	●		
Laboratory procedures								
Maternal rapid HIV ^a^	●							
Maternal pregnancy test^a^	●							
Maternal rapid syphilis ^a^	●							
Maternal candida, gram stain^a^	●							
Maternal hemoglobin (HemoCue)^a^	●				●			
Maternal urinalysis^a^	●				●			
Maternal viral load and T cell assays	●		●				●	
Placenta, membranes, cord blood storage							●	
Infant HIV DNA PCR^a^								●
Vaginal-rectal swab storage	●		●	●			●	
Blood storage	●		●	●			●	
Urine storage	●		●	●			●	

^a^Performed as standard antenatal care

^b^Occurs weekly through week 36, or until delivery, whichever occurs first

^c^All procedures may not be completed for women who deliver off-hours or in a location without staff coverage

Participants then undergo randomization into one of two treatment groups and start study drug as early as possible within the window of 16 and 24 weeks of gestation. Eligible women who choose to participate at the screening visit are given a subsequent appointment for randomization no earlier than the following business day to encourage careful consideration of the risks and benefits of study participation. On the day of randomization, following final confirmation of eligibility, participants are assigned with equal probability into one of two study groups. Our randomization scheme stratifies participants by timing of ART initiation (during current pregnancy / pre-pregnancy). A statistician from the UNC Center for AIDS Research Biostatistics Core not otherwise associated with the study created the scheme using random permuted blocks. At the time of randomization, a research nurse confirms eligibility in a web-based randomization program. Using the same web-based tool, the pharmacist then performs the randomization procedure and prepares the 1 mL injection of the assigned study treatment, which the research nurse administers to the participant. All randomization documentation is stored securely.

**Fig. 2:**
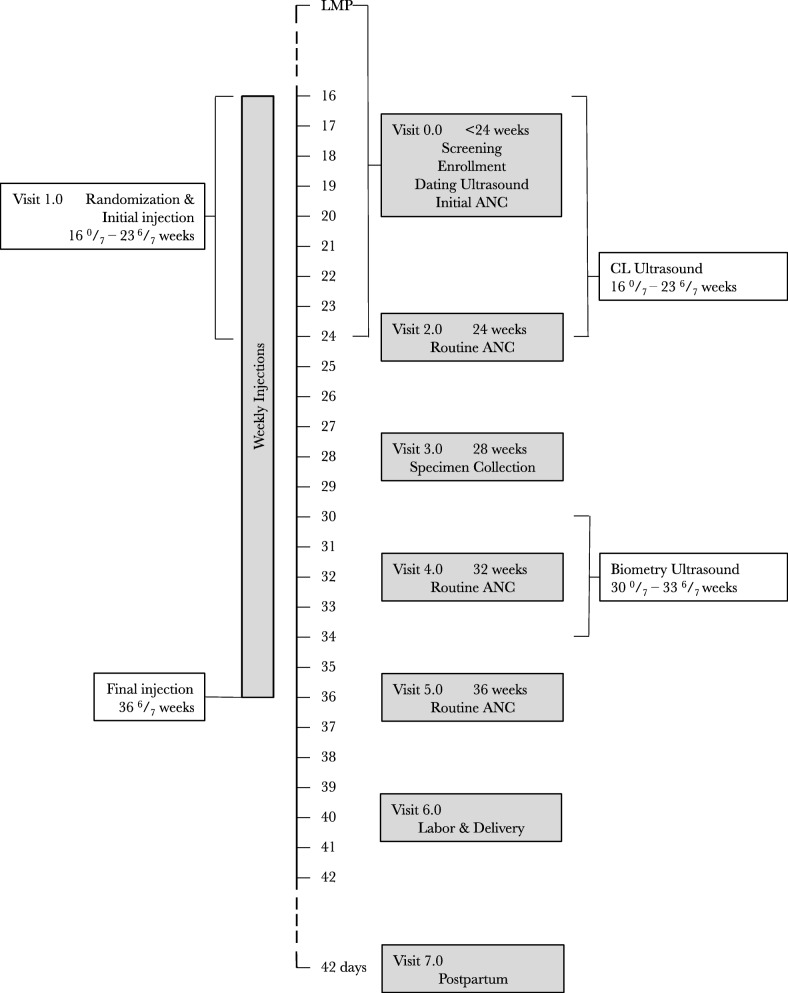
IPOP study trial schema

Once IPOP participants start study drug, they are seen weekly (Fig. 2). At each injection visit, study staff evaluate participants for side effects of IM progesterone, which are expected to be rare and generally mild, but include injection-site reactions, headache, breast tenderness, nausea, and cough. In addition, the study provides routine antenatal care to all participants following Zambian national guidelines at: 24, 32, and 36 gestational weeks.

At the 24- and 28-week study visits, blood, urine, and vaginal swabs are collected. At the time of delivery or shortly thereafter, blood, urine, and vaginorectal swabs are collected (Table 1). At delivery, study nurses collect samples of the placenta, umbilical cord, and cord blood. Also at delivery, study nurses obtain detailed information about the participant and her infant’s clinical course by patient self-report, review of medical records, or by direct assessment. We assign a clinical phenotype [17] for all PTB and stillbirth outcomes as soon as possible following delivery. Finally, we conduct a routine 42-day postnatal visit, which includes clinical assessment of both mother and infant, and testing for infant HIV infection.

### Laboratory procedures

Study staff obtain all samples from trial participants according to standard operating procedures. All samples are processed according to the assay manufacturers’ specifications. Some specimens collected from patients in this protocol are analyzed immediately per standard antenatal care guidelines. Others are stored temporarily for later study-related analysis to investigate biological mechanisms contributing to preterm birth and other adverse birth outcomes.

### Study monitoring

An external monitor will be contracted to oversee the progress of the clinical trial and to ensure that it is conducted, recorded, and reported in accordance with the protocol, standard operating procedures, International Conference on Harmonization / Good Clinical Practices, the Zambian National Health Research Act, and U.S. 45 CFR 46 requirements. The monitor will verify that the data reported is valid, accurate, and complete through review of case report forms, medical records, and source documentation. The monitor will make site visits each quarter through the end of the study and will provide verbal and written feedback to the study site for ongoing quality control and improvement.

We will constitute a Data Safety and Monitoring Board (DSMB) to periodically monitor trial performance and safety. The group will comprise senior investigators with statistical, methodological, and topical expertise who are not otherwise involved with the study. Their first charge will be to review the study protocol and create a monitoring schedule and stopping guidelines, based on expected event rates. Other specific duties will include: (1) periodic assessments of recruitment, accrual, retention, and data quality; (2) considering new external data as they come available, including scientific or therapeutic developments that may have an impact on participant safety or the ethics of the trial; (3) evaluation of scheduled interim analysis. Mortality outcomes will be reviewed by the DSMB during routine meetings. The DSMB will have authority to enjoin enrolment or stop the study altogether for reasons of patient safety. If an interim analysis were to show unequivocal benefit before follow-up were complete, we would support stopping the study and reprogramming remaining resources to make the intervention available to all participants.

### Primary outcome

Our primary outcome is a composite of live birth occurring before 37 gestational weeks or stillbirth at any gestational age. Secondary outcomes are: delivery prior to 34 weeks of gestation and prior to 28 weeks of gestation; birth weight < 10th percentile for gestational age; birth weight < 3rd percentile for gestational age; mother-to-child HIV transmission by 6 weeks postpartum; cumulative incidences of the competing risks of stillbirth and PTB; neonatal and perinatal mortality rates; infant APGAR scores. Our tertiary outcomes are: birth weight < 2500 g and < 1500 g; measures of maternal inflammation and/or immune activation; alterations of the vaginal microbiome; measures of infant morbidity (e.g., intensive care unit admission, supplemental oxygen requirement, need for assisted ventilation); and serious adverse events and events resulting in study product discontinuation.

### Sample size

We estimated the baseline risk of the composite outcome (p1) as 24%, based on estimates of preterm birth (21%) plus stillbirth (3%) among HIV-infected women in Zambia from our own local data [18, 19] and from modeled data [2]. We hypothesize that 17P will reduce the proportion of HIV-infected women experiencing the composite endpoint by 38% (from 24 to 15%) [12]. A trial with 325 patients per group would have 80% power to detect this effect size (based on a 2-sided Fisher’s exact test at the 0.05 significance level). To allow for up to 15% follow-up loss and to account for uncertainty in our estimates of baseline event probability (p1) and relative efficacy of the intervention, we set target enrollment at 400 women per randomization group in the trial.

### Statistical analysis plan

We will assign an estimated delivery date (EDD) by ultrasound biometry measurements collected at time of enrollment, using the INTERGROWTH 21st standards [20, 21]. We do not consider the participants’ reported last menstrual period in our gestational age calculations, as this has been shown in our setting to introduce bias [22]. Randomized women who deliver (1) a live infant 21 or more days before their assigned EDD or (2) a stillborn infant at any gestational age will be categorized as having met the primary outcome.

The primary analysis will employ the intent-to-treat principle, wherein each mother-infant pair will be analyzed according to randomization assignment regardless of whether the mother complied with study procedures. The primary comparison between the two trial groups will be based on the estimated risk ratio of the primary outcome. The estimated risk of the primary outcome will be computed in each group using the Kaplan-Meier method to adjust for loss-to-follow-up, where mother-infant pairs who drop out of the trial prior to the primary outcome or live-birth of full-term infant will be right censored at their last study visit. A *Z*-statistic will be constructed by taking the difference in the estimated log risks divided by the corresponding estimated standard error (using the Greenwood estimator). As a sensitivity analysis, a Cox proportional hazards model with treatment assignment as the sole covariate will be fit to estimate the hazard of meeting the primary endpoint between randomization groups; the estimated hazard ratio is anticipated to be similar to the estimated risk ratio given that the primary outcome is relatively rare.

We plan to undertake a pre-specified subgroup analysis for this trial. This subgroup analysis will assess whether the effect of 17P differs within levels of the stratification factor. Effect modification will be assessed by fitting Cox proportional hazards models that include randomization group, the stratification factor, and a product term as covariates. A likelihood ratio test of the product term coefficient will be used to assess possible effect modification. If the effect modification tests are significant, hazard ratios will be estimated separately within levels of the factor (e.g., hazard ratios of 17P versus placebo for (i) mothers on pre-pregnancy ART and (ii) mothers initiating ART in pregnancy).

To assess the efficacy of 17P, a per-protocol analysis of the primary outcome will also be conducted that censors participants who fail to comply with their randomization assignment (defined here as missing two injections of study product in a row), and then reweights the data to account for possible informative censoring by noncompliance using inverse-probability (IP) weights [23]. We have previously used this and related methods to construct a consistent estimator of the per-protocol effect under the assumption that we have measured and correctly-adjusted for the common causes of noncompliance and the outcome [24–26]. The set of measured possible common causes includes demographic, medical, and obstetrical factors. We will construct IP weights using pooled logistic regression (pooling over time), with continuous variables fit using flexible restricted splines. We will explore the sensitivity to IP weight estimation [27].

### Ethical considerations

Participation in this trial is voluntary. All participants undergo a comprehensive written informed consent process prior to study enrollment. Clinical study procedures are conducted according to local standards of routine care. All staff with direct participant contact receive training on protecting human research participants [28] before performing any study procedures and routinely thereafter. Key staff also undergo Good Clinical Practices training [29] no less frequently than every 3 years. The University of Zambia Biomedical Research Ethics Committee and the University of North Carolina at Chapel Hill Institutional Review Board each granted approval of the IPOP trial protocol before enrollment commenced.

Investigators make every reasonable effort to minimize risks to participants. We expect that participants will be exposed to minimal risk in this trial. Side effects and serious adverse events associated with 17P administration have been studied extensively and are rare [30]. Women with one or more contraindication to 17P that appear in the Makena® package insert [31] are excluded from the trial. Physical risks of IPOP study participation also include the risks associated with venipuncture, which are infrequent and minimized with the use of proper technique. Collection of vaginal and rectal samples may also be associated with some discomfort or mild bleeding.

Participation in clinical research includes possible breach of confidentiality as well as discomfort with personal medical and socio-behavioral questions. Although investigators make every effort to protect participant privacy and confidentiality to reduce these risks (e.g., by conducting consent procedures in a private setting and do not include participant names on case report forms), it is possible that participant involvement in the study could become known to others, and that social harms may result (e.g., participants could become known as HIV-infected).

At each study visit, study staff assess participants for occurrence of social harms and adverse events. The severity of any study-related adverse events is graded based on the National Institute of Health’s Division of AIDS Table for Grading the Severity of Adult and Pediatric Adverse Events [32]. Study staff report adverse events or social harms that are related to the study drug and/or study participation according to requirements by each individual regulatory authority.

To maintain participant confidentiality, all laboratory specimens, reports, study data, and administrative forms are identified by a coded number only. All databases are secured with password-protected access systems, and computer entries are identified by coded number only. Forms, lists, logbooks, appointment books, and any other listings or data forms that link participant identification numbers to other identifying information are stored in a separate, locked cabinet. All statistical analyses will use data identified only by the coded study number. Clinical information with individual identifiers will not be released without the written permission of the participant.

Participants who receive 17P in the IPOP trial may benefit from a reduced risk of delivering preterm if the intervention is indeed effective, but it is also possible that there will be no effect. Participants in both study groups may benefit from added health education and counseling, comprehensive antenatal care, and close follow-up. Findings from this study could shape health policy and clinical care, or inform future research in the prevention of HIV-related PTB. We intend to make all learning from this trial widely and quickly available by publishing in open access journals to facilitate informed decision-making by key stakeholders engaged in PTB prevention worldwide.

## Discussion

The global epidemics of preterm birth and HIV converge in sub-Saharan Africa, where the skilled providers and resources needed to care for preterm neonates are scarce. Antiretroviral therapy for the prevention of mother-to-child HIV transmission has led to encouraging reductions in new cases of pediatric HIV, but despite preventing transmission this treatment does not appear to reduce the elevated risk of PTB and neonatal morbidity/mortality compared to HIV-uninfected pregnant women; in a study in Botswana 23% of women living with HIV on ART experience PTB compared to 15% of uninfected women [10]. In resource-limited settings where HIV complicates a considerable proportion of pregnancies, strategies to ameliorate PTB risk in women using ART could result in a substantial public health impact.

With this Phase III double-masked, placebo-controlled, randomized clinical trial, we aim to discover whether 17P will prevent PTB and stillbirth among HIV-infected women receiving ART in pregnancy. We will also, through planned sub-group analysis, gain insight into whether the effect of 17P varies between women initiating antiretroviral therapy prior pregnancy versus those newly starting treatment in pregnancy. Although pilot studies of vaginal progesterone to prevent PTB among HIV-infected women are ongoing [33, 34], the IPOP Study represents the first full-scale efficacy trial of antenatal progesterone in this population. If efficacious in this high-risk group, 17P has the potential to prevent hundreds of thousands of preterm births per year worldwide.

## Abbreviations

17P: 17-alpha hydroxyprogesterone caproate
ANC: antenatal care
ART: Antiretroviral therapy
CONSORT: Consolidated Standards of Reporting Trials
DSMB: Data Safety and Monitoring Board
EDD: Estimated delivery date
HIV: Human immunodeficiency virus
IM: Intramuscular
IP: inverse probability
IPOP: Improving pregnancy outcomes with progesterone
KDHC: Kamwala District Health Centre
LMP: last menstrual period
PTB: Preterm birth
SPIRIT: Standard Protocol Items: Recommendations for Interventional Trials
UNC: University of North Carolina
WNH-UTH: Women and Newborn Hospital of the University Teaching Hospital
